# Academic Performance of Native and Immigrant Students: A Study Focused on the Perception of Family Support and Control, School Satisfaction, and Learning Environment

**DOI:** 10.3389/fpsyg.2016.01560

**Published:** 2016-10-13

**Authors:** Miguel A. Santos, Agustín Godás, María J. Ferraces, Mar Lorenzo

**Affiliations:** ^1^Faculty of Education, University of Santiago de CompostelaA Coruña, Spain; ^2^Faculty of Psychology, University of Santiago de CompostelaA Coruña, Spain

**Keywords:** academic performance, family support and control, school satisfaction, learning environment, immigrant students

## Abstract

The international assessment studies of key competences, such as the PISA report of the OECD, have revealed that the academic performance of Spanish students is significantly below the OECD average. In addition, it has also been confirmed that the results of immigrant students are consistently lower than those of their native counterparts. Given the context, the first objective of this work is to observe the variables (support, control, school satisfaction, and learning environment) which distinguish between retained and non-retained native and immigrant students. The second objective is to check, by comparing the retained and non-retained native and immigrant students and separating the two levels, in order to find out which of the selected variables clearly differentiate the two groups. A sample of 1359 students was used (79.8% native students and 20.2% immigrant students of Latin American origin), who were enrolled in the 5th and 6th year of Primary Education (aged 10–11 years) and in the 1st and 2nd year of Secondary Education (aged 12–13 years). The measurement scales, which undergo a psychometric analysis in the current work, have been developed in a previous research study (Lorenzo et al., 2009). The construct validity and reliability are reported (obtaining alpha indices between 0.705 and 0.787). Subsequently, and depending on the results of this analysis, inferential analyses are performed, using as independent variables the ethno-cultural origin and being retained or not, whereas, as dependent variables, the indices referring to students' perception of family support and control, as well as the assessment of the school and learning environment. Among other results, the Group × Being retained/Not being retained [*F*_(1, 1315)_ = 4.67, *p* < 0.01] interaction should be pointed out, indicating that native non-retained subjects perceive more control than immigrants, as well as the Group × Being retained/Not being retained [*F*_(1, 1200)_ = 5.49, *p* < 0.01] interaction, showing that native non-retained students perceive more family support. Given the results obtained, our intention is to provide solid evidence that would facilitate the design of family involvement programs, helping to improve students' educational performance.

## Introduction

Although, as a result of the economic crisis, the number of immigrants arriving in Spain has diminished in recent years, and in spite of the warnings that immigration flows are increasing in most countries (OECD, 2015), we should not lose sight of two important elements that characterize Spanish migratory flows: first, the quantitative data that, at the end of 2015, there were a total of 4,905,495 foreigners residing in Spain; and secondly, with a constant influx of immigrants in the twenty-first century, there is an increased presence of family immigration which results in a significant number of children from these families being attendant within the Spanish educational system.

More specifically, in Spain the figure for the academic year 2014–2015 showed 712,098 foreign children (8.8% of the total), mainly from African countries (30.47%) and the EU-28 (27.49%), who were primarily enrolled in Secondary Compulsory Education (Ministry of Education Culture, and Sports, 2015). These students' school success is obviously essential for their social inclusion.

Data from the latest PISA Report (2012) of the OECD, show that the academic performance of Spanish students remains basically stable in relation to previous editions, that is, it is still significantly below the OECD average (Ministry of Education Culture, and Sports, 2013). In this context, it is not surprising that the early dropout rate in Spain is twice the average, and is on an upward trend, in contrast to other European countries (Casquero et al., 2012).

But the Report also reflects an even less flattering reality for the students of immigrant origin living in Spain. The assessment of competences shows that the results of these students are consistently lower than those obtained by native students. More specifically, in Mathematics, students of immigrant origin obtained a mean of 439.1 points compared to 491.7 points obtained by their Spanish counterparts. In any case, the mean improves (457) with second-generation students (students born in Spain with both parents of foreign origin; Calero and Escardibúl, 2013).

Similar conclusions were drawn by Vaquera and Kao (2012) in their study conducted with a sample of 2710 Compulsory Secondary Education students. These authors confirm the constant disadvantage regarding the performance of first-generation immigrant students in Spain, with Latin American students showing the lowest performance overall.

This trend can be extrapolated to other countries, as reflected in the scientific literature. In particular, Schnell and Azzolini (2015) focus their research, based on PISA 2009 and 2012, on countries from southern Europe (Greece, Spain, Portugal, and Italy), which share the status of being recent immigration destinations.

These authors state that there are big gaps in terms of educational achievement between immigrant and native children in these four countries. Their results also suggest the existence of a negative association, although weak, between the age of arrival in the host country and their school performance. If immigrant children arrive after 6 years of age (when Compulsory Education begins), they face the greatest disadvantages in terms of educational performance, whereas the second-generation students and those who arrive at an early age perform, on average, better than the former, even if they do not reach the native students' level.

López et al. (2001) stated that migrants were academically the most vulnerable group in the United States, showing lower academic performance and higher dropout rates. In this regard, Levels et al. (2008) explained how the results of these students should be interpreted according to their country of origin and destination, showing that these students have a better educational performance in countries traditionally known as immigrant destinations. This is corroborated, contrary to what most studies argue, by Areepattamannil et al. (2015) who found differences in favor of first- and second-generation immigrant adolescents, compared with their counterparts in Qatar, in terms of performance and disposition toward Mathematics.

Considering the data above, it is not surprising that school failure, one of whose determining factors is precisely retention, is one of the major problems of the Spanish education system, given its magnitude, evolution, and social consequences (OECD, 2012).

Social research has attempted to identify the variables that explain students' performance, and even the differences that occur between native and immigrant students. Thus, it is argued that performance is influenced by both a number of factors and the interaction between these factors (Barbero et al., 2007; Creemers and Kyriakides, 2008; Winne and Nesbit, 2010). Notably, the circumstances in which learning is developed, the starting conditions, as well as the social, economic and cultural backgrounds of students and schools should be taken into account (Suárez-Orozco and Suárez-Orozco, 2008; Lorenzo et al., 2009, 2012; Suárez et al., 2011).

The research provides a greater explanatory weight to students' individual variables, with the socioeconomic and cultural background of the family being of particular importance (Eccles, 2005; Grayson, 2011); by contrast, it also provides a reduced weight to center variables, such as school characteristics, its resources, educational processes, or composition of its student body (Santos Rego et al., 2012, 2013; Calero and Escardibúl, 2013). From a comparative perspective, the weight of the students' variables is more pronounced in the Spanish context (Cordero et al., 2013).

On the same line of research, the results of the study conducted by Núñez et al. (2014) with upper-secondary education students from Spain and Portugal, showed that most (85.6%) of the observed performance variability in the subject of Biology, is due to students' variables, whereas only the remaining 14.4% corresponds to classroom-related variables.

Specifically, at student level, performance was found to be associated with the learning approach, prior knowledge, school absenteeism, and parents' educational level. At classroom level, performance is only associated with the teachers' teaching approach, although not associated directly, but through students' own study approach. It should be recalled, in this sense, that the Coleman Report in 1966 had already attributed 10% of the students' performance variance to the school, whereas the remaining 90% had been attributed to students' socioeconomic status (Coleman et al., 1966).

Han (2006) argued that the characteristics of the child and their family accounted for many of the differences in academic achievement of immigrant children, whereas their home, school, and neighborhood, although important, are not as pronounced. In any case, the home and school influence on performance is higher for Latin American children than for those of Asian origin.

At present, the research on the determining factors of academic performance continues to seek evidence in connection with the student's personal motives (Carbonero et al., 2015), in the weight of the socioeconomic status of the families that support them, and a number of contextual determinants, many of which still need to be determined.

The study presented herein is based on the structure of the dynamic model developed by Creemers and Kyriakides (2008), which establishes a set of related factors, grouped on four levels to explain educational effectiveness: the contextual level, which includes national or regional education policies and an evaluation thereof; the school level, which covers the analysis and evaluation of both the educational project of each center and its planning with respect to the learning environment; the classroom level, which is based on an analysis of the faculty's guidance when pointing out targets for the specific content to be explained, of the materials, the techniques used to encourage discussion, strategies to solve the designed activities and the opportunities to implement or apply the explained content; and finally, regarding the students' level, other factors are proposed.

On the one hand, the so-called stable factors (family's socioeconomic status, ethnicity, personality traits, and gender), and, on the other, factors which can change over time, including expectations, motivation, and thinking styles. Other, more psychological factors should not be omitted either: skills, perseverance, and variables related to specific learning tasks, that is, time devoted to homework and learning opportunities.

## Aims of this study

The research line followed in this work has been previously considered with other populations and other variables, both individual and contextual (Covington, 2000; González-Pienda et al., 2003; Valle et al., 2009; Barca et al., 2012; Santos Rego et al., 2012, 2013; Dufur et al., 2013; Núñez et al., 2014). These works found that the highest average academic performance (in terms of specific grades) is based on student's personal characteristics, motivational variables, support and family control and friendly relationships. All of them, with uneven influential weight, determine satisfactory or unsatisfactory response in terms of the school context and set of variables: that is, they determine the best or worse academic performance.

Given the context, our first objective is to observe what the variables are regarding family support and control, school satisfaction, and assessment of the learning environment that distinguish between retained and non-retained native and immigrant students; the second objective is to check, by comparing the retained and non-retained native and immigrant students, and separating the two levels, in order to find out which of the selected variables clearly differentiate the two groups.

## Materials and methods

### Participants

The study involved a total of 1359 individuals enrolled in the last 2 years (5th and 6th) of Primary Education (42.3%) and the first two (1st and 2nd) of Secondary Education (57.7%), from 33 schools selected according to the official statistics of educational administration, based on two criteria: the first one was that they taught the two levels of education; and the second was that a large number of immigrant students be enrolled in the respective school and academic years. Thus, 79.8% of the selected individuals are Spanish (native students) and 20.2% have a Latin American origin (immigrant students). Table 1 shows the main sociodemographic characteristics.

**Table 1:** **Characteristics of the individuals participating in the research study (%)**.

**Variables**	**Native students**	**Immigrant students**
**GENDER**		
Males	80.1	79.5
Females	19.9	20.5
**AGE**		
9–10 years old	17.4	10.4
11–12 years old	51.9	44.9
13–14 years old	26.7	35.6
15–16 years old	3.9	9.6
**GRADE**		
5th Primary Education	12.3	8.0
6th Primary Education	42.5	41.6
1st Secondary Education	28.2	33.6
2nd Secondary Education	17.0	16.8
**FAMILY COMPOSITION**		
Father and mother	71.7	55.5
Father	1.3	0.4
Mother	6.5	11.3
Father and relatives	3.0	6.6
Mother and relatives	7.8	16.4
Father and other people	0.3	0
Mother and other people	0	0.4
Other relatives	8.0	8.3
Other people	1.4	1.1
**NUMBER OF SIBLINGS**		
None	16.6	9.1
One	59.7	30.3
Two	15.8	27.3
Three	4.8	17.2
More than three	3.1	16.1

### Measures

A single instrument (questionnaire) was used, consisting of closed and categorical questions regarding students' socio-demographic profile (see Table 1) and three *Likert* scales, on the perception of family support and control and on the overall assessment of the school and learning environment, comprising a total of 43 items. The three scales were developed and used at a descriptive and inferential level in a research project aimed at evaluating an educational intervention program by Lorenzo et al. (2009).

#### Family support scale

This consists of 14 items (with 7 each referring to the father and mother) relating to support in terms of mood, help when facing problems, assistance with school work, perception of trust, respect, concern, and clear communication of expectations. Five-response choices were used—1: never, 2: rarely; 3: sometimes; 4: almost always, 5: always. Its factorial structure and reliability indices can be considered coherent and acceptable, as shown in Table 2, with the following values: the general α index was 0.854, the paternal support α index was 0.842, and the maternal support α index was 0.705.

**Table 2:** **Factorial structure of the control scales (FC-fathers and MC-mothers), family support (FS-fathers and MS-mothers), and assessment of the school environment, saturation values and reliability index (Alpha index)**.

**Control. general**	**Support. general**	**Assessment of the school. general**
KMO = 0.771 Chi-square = 7559.498; *p* ≤ 0.000.	KMO = 0.768 Chi-square = 9410.088; *p* ≤ 0.000.	KMO = 0.925 Chi-square = 6434.421; *p* ≤ 0.000.
Explained variance (69.244%).	Explained variance (73.758%).	Explained variance (58.823%).
Factor I (38.679%). Items: FC (0.741) and MC (0.694) about friends. FC (0.686) and MC (0.667) about their behavior on the street. FC (0.643) and MC (0.614) about their timetable.	Factor I (35.408%). Items: FS (0.767) encouraged to do the best they can. FS (0.743) help when facing problems. FS (0.733) help with school work.	Factor I (34.399%) Items: My parents' reaction to my grades (0.790). The image that my teachers have of me (0.734). Evaluations (0.575). Relationships with teachers (0.549).
Factor II (11.964%). Items: MC (0.909) and FC (0.818) about money.	Factor II (11.056%). Items: FS (0.839) and MS (0.478) respect me. MS (0.686) and FS (0.656) I trust them.	Factor II (7.131%). Items: How rules are enforced (0.789). Penalties for breaking rules (0.735).
Factor III (9.922%). Items: MC (0.867) and FC (0.863) over their attendance at school.	Factor III (9.496%). Items: MS (0.818) encouraged to do the best they can. MS (0.762) help when facing problems. MS (0.415) help with school work.	Factor III (6.059%). Items: We work hard as a group (0.728). They let us make decisions about the tasks (0.695). Public recognition from the teacher (0.505). Classes are interesting and varied (0.430).
Factor IV (8.678%). Items: FC (0.834) and MC (0.783) over the hours devoted to daily study.	Factor IV (9.057%). Items: MS (0.937) and FS (0.856) clear communication of expectations.	Factor IV (5.721%). Items: Teachers want us to understand (0.811). Teachers want us to enjoy learning (0.659). We have enough time for homework (.470).
	Factor V (8.741%). Items: MS (0.904) and FS (0.695) care about my academic success.	Factor V (5.513%). Items: With the peers (0.932) With the school in general (0.405).
Reliability indices:	Reliability indices:	Reliability indices:
General alpha index = 0.855	General alpha index = 0.854	General alpha index = 0.871
Paternal control alpha index = 0.793	Paternal support alpha index = 0.842	Satisfaction alpha index = 0.827
Maternal control alpha index = 0.725.	Maternal support alpha index = 0.705.	Learning environment alpha index = 0.782.

#### Family control scale

This consists of 12 items (with 6 each referring to the father and mother) on the control over the time their children spend away from home, their friends, their activities outside the home, how they spend their money, school attendance and time spent each day to study. Five-response choices were used—1: none, 2: a little, 3: somewhat; 4: quite a lot; 5: very much—and its factorial structure, as well as its reliability indices are satisfactory and consistent with the objective of the instrument (see Table 2). In this case, the general α index was 0.855, the paternal control α index was 0.793, and the maternal control α index was 0.725, respectively.

#### School environment rating scale

The third scale consists of 17 items, 9 of them on school satisfaction and 8 on assessment of the learning environment:

1. The subscale of satisfaction collects information on personal relationships with teachers, subjects, forms of evaluation, relationships with peers, the image that teachers have about students, parents' reaction to the grades obtained, the school they are enrolled in, how to meet the school's standards, and the sanctions imposed when failing to comply with them. Responses include five options—1, very satisfied; 2, fairly satisfied; 3, indifferent; 4, quite dissatisfied; 5, very dissatisfied.
2. The assessment subscale of the learning environment provides information about teachers' teaching styles, ways of reinforcing students, the public recognition of a job well done, the perception of fairness in assessments, the use of group work, sufficient time available for homework, promotion of autonomy in students, and the general evaluation of the courses received. The response format is similar to the satisfaction scale with five response options—1, strongly agree; 2, somewhat agree; 3, indifferent; 4, somewhat disagree; 5, strongly disagree.

Both the factorial structure and the reliability indices of this third scale can be considered acceptable for the goals set out in this study (see Table 2). The general α index was 0.871, the satisfaction α index was 0.827, and the learning environment α index was 0.782.

### Procedure

For the application of the questionnaire, which is anonymous, the educational authorities were instructed to initially request permission, and subsequently the families were informed. The questionnaire was administered collectively in the classroom, using tutors of each group that were especially trained, not only for this task, but also within the framework of a broader data collection for an educational research project.

### Data analysis

As independent variables, (native or immigrant) students' ethno-cultural background and being a retained student or not are used. The choice of the latter is given by the powerful connection between being a retained student and obtaining low academic results in two subjects: Mathematics and Spanish Language and Literature (see Table 3). As dependent variables, their perception of support and control by parents, their school satisfaction and their assessment of the learning environment are taken into account.

**Table 3:** **Differentiation rates for native^a^ and immigrant^b^ students and percentage of students non-retained (NR) and retained (R) in Mathematics and Spanish Language and Literature**.

	**Chi-Square**	***df***	***p***	**%fe ≤ 5**	**Cramer's V**	**NR (%)**	**R (%)**
Mathematics	222.943^a^	1	0.001	0	0.468^a^	90.1^a^	9.9^a^
	28.806^b^	1	0.001	0	0.333^b^	69.2^b^	30.8^b^
Spanish language and literature	222.943^a^	1	0.001	0	0.477^a^	90.3^a^	9.7^a^
	28.806^b^	1	0.001	0	0.440^b^	75.0^b^	25.0^b^

Descriptive analyses were conducted using the sociodemographic variables. Next, three exploratory factor analyses (EFA) were carried out in order to understand the factorial structure and reliability of the used scales. In addition, the differences among the participating groups were analyzed using ANOVA, in which the independent variables related to being native or immigrant, and being a retained student or not. The dependent variables are the perception of family control and support, and the assessment made by the students of their school environment. Finally, two separate logistic regression analyses (native/immigrant) were performed with the aim of studying which variables can predict whether a student will be retained or not.

## Results

### Analysis of differences with respect to family control

In this dimension (see Table 4), there are significant differences on the four indices, with different results in each of them. In the first index (C1), which refers to the “paternal and maternal control over the behavior outside the home,” significant differences on the two factors were recorded, which is not the case when analyzing the interaction. In pairwise comparisons, significant differences were found with regard to being retained, both in native (M_retained − Non_retained = −2.69, *p* < 0.001), and immigrant students (M_retained − Non_retained = −1.99, *p* < 0.01). In this index, both native and immigrant non-retained students are those who perceived a greater control of their behavior, by their parents, when outside the home.

**Table 4:** **Results of the analysis of variance (ANOVA) for variables related to maternal and paternal control (NS-Native students; IS-Immigrant students)**.

**Factors**	**Ethno-cultural origin**		**Being retained**		**Interaction**		**Pairwise comparison**
	**Mean**	***F***	**Mean**	***F***	**Mean**	***F***	**Difference of means**
C1	NS (22.08)	*F*_(1, 1315)_ = 7.81^**^	No (22.49)	*F*_(1, 1315)_ = 27.87^***^	NS-No (22.75)	*F*_(1, 1315)_ = 0.61 (ny)	NS No-Yes = 2.69^***^
	IS (20.07)	η^2^ = 0.013, 1−β = 0.92	Yes (19.77)	η^2^ = 0.021, 1−β = 1.00	NS-Yes(20.05)		IS No-Yes = 1.99^***^
					IS-No (21.15)		
					IS-Yes (19.16)		
C2	NS (7.06)	*F*_(1, 1315)_ = 12.86^***^	No (7.15)	*F*_(1, 1315)_ = 11.11^**^	NS-No (7.33)	*F*_(1, 1315)_ = 4.67^**^	NS No-Yes = 1.08^**^
	IS (6.09)	η^2^ = 0.01, 1−β = 0.95	Yes (6.15)	η^2^ = 0.008, 1−β = 0.91	NS-Yes (6.25)	η^2^ = 0.004, 1−β = 0.58	
					IS-No (6.20)		
					IS-Yes (5.97)		
C3	NS (8.81)	*F*_(1, 1315)_ = 9.75^**^	No (8.91)	*F*_(1, 1315)_ = 15.32^***^	NS-No (9.01)	*F*_(1, 1315)_ = 1.47 ny	NS No-Yes = 0.81^***^
	IS (8.15)	η^2^ = 0.007, 1−β = 0.88	Yes (8.11)	η^2^ = 0.012, 1−β = 0.97	NS-Yes (8.20)		
					IS-No (8.33)		
					IS- Yes (7.90)		
C4	NS (7.99)	*F*_(1, 1315)_ = 3.00 ny	No (8.18)	*F*_(1, 1315)_ = 30.82^***^	NS-No (8.26)	*F*_(1, 1315)_ = 1.33 ny	NS No-Yes = 1.09^***^
	IS (7.47)		Yes (7.12)	η^2^ = 0.022, 1−β = 1.00	NS-Yes (7.17)		
					IS-No (7.79)		
					IS-Yes (7.07)		

^*^p < 0.05,

** *p < 0.01*,

*** *p < 0.001*.

Significant differences were also observed in both factors and the interaction [*F*_(1, 1315)_ = 4.67, *p* < 0.01] in the second index (C2) “father and mother's control over their children's money.” This indicates that there are differences between native retained and non-retained students [*F*_(1, 1315)_ = 1.08, *p* < 0.01], but this is not the case with immigrant students when observing the pairwise comparison. Nevertheless, those who have a better academic performance are more aware of their parents' control over their money.

The third index (C3) relating to “paternal and maternal control over their attendance at school,” also reported significant main differences [ethno-cultural origin, *F*_(1, 1315)_ = 9.75, *p* < 0.01; being a retained student, *F*_(1, 1315)_ = 15.32, *p* < 0.001], but the interaction is not significant. In the pairwise comparison (M_Non-retained − M_retained = 81, *p* < 0.001) differences were recorded only between native retained and non-retained students, with the latter perceiving the paternal and maternal control over their school attendance more intensely.

In the last index (C4), referring to the “paternal and maternal control over the hours devoted to daily study,” significant differences only refer to being a retained student or not [*F*_(1, 1315)_ = 30.82; *p* < 0.001]. In this case, the native students are those who acutely felt their parents control in this aspect (M_Non-retained − M_retained = 1.09, *p* < 0.001).

### Analysis of differences with respect to family support

The first thing to highlight (see Table 5) is the lack of significant differences in the five indices of family support, in the “ethno-cultural origin” (native or immigrant) factor.

**Table 5:** **Results of the analysis of variance (ANOVA) for variables related to maternal and paternal support (NS, Native students; IS, Immigrant students)**.

**Factors**	**Ethno-cultural origin**		**Being retained**		**Interaction**		**Pairwise comparison**
	**Mean**	***F***	**Mean**	***F***	**Mean**	***F***	**Difference of means**
PS1	NS (16.57)	*F*_(1, 1200)_ = 0.94 ny	No (16.82)	*F*_(1, 1200)_ = 11.72^**^	NS-No (16.97)	*F*_(1, 1200)_ = 5.49^**^	NS No-Yes = 1.71^**^
	IS (15.84)		Yes (15.37)	η^2^ = 0.010, 1−β = 0.93	NS-Yes (15.25)	η^2^ = 0.005, 1−β = 0.65	IS No-Yes = 0.35 ny
					IS-No (15.98)		
					IS-Yes (15.66)		
PS2	NS (17.33)	*F*_(1, 1200)_ = 0.06 ny	No (17.72)	*F*_(1, 1200)_ = 34.33^***^	NS-No (17.65)	*F*_(1, 1200)_ = 1.40 ny	NS No-Yes = 1.82^***^
	IS (17.00)		Yes (16.05)	η^2^ = 0.0228, 1−β = 1.00	NS-Yes (15.94)		IS No-Yes = 1.21^**^
					IS-No (17.51)		
					IS-Yes (16.30)		
PS3	NS (17.50)	*F*_(1, 1200)_ = 1.86 ny	No (17.67)	*F*_(1, 1200)_ = 10.55^**^	NS-No (17.76)	*F*_(1, 1200)_ = 12.84 ny	NS No-Yes = 1.26^***^
	IS (16.91)		Yes (16.65)	η^2^ = 0.009, 1−β = 0.90	NS-Yes (16.63)		IS No-Yes = 0.35 ny
					IS-No (17.06)		
					IS-Yes (16.70)		
PS4	NS (8.44)	*F*_(1, 1200)_ = 0.65 ny	No (8.54)	*F*_(1, 1200)_ = 3.97 ny	NS-No (8.57)	*F*_(1, 1200)_ = 2.70 ny	NS No-Yes = 0.57^***^
	IS (8.36)		Yes (8.10)		NS-Yes (8.00)		IS No-Yes = 0.03 ny
					IS-No (8.37)		
					IS-Yes (8.34)		
PS5	NS (9.33)	*F*_(1, 1200)_ = 0.40 ny	No (9.49)	*F*_(1, 1200)_ = 21.92^***^	NS-No (9.52)	*F*_(1, 1200)_ = 2.17 ny	NS No-Yes = 0.76^***^
	IS (9.16)		Yes (8.80)	η^2^ = 0.018, 1−β = 0.99	NS-Yes (8.75)		IS No-Yes = 0.39 ny
					IS-No (9.33)		
					IS-Yes (8.94)		

^*^p < 0.05,

** *p < 0.01*,

*** *p < 0.001*.

However, a significant difference on the factor of being a retained student or not was observed, as well as some pairwise comparisons which should be pointed out.

As for the PS1 index corresponding to the perception of “paternal support and encouragement when dealing with both school work and problematic situations,” the significative differences lies with the non-retained students [*F*_(1, 1200)_ = 11.72; *p* < 0.01]. Similarly, significant differences were observed in the interaction [*F*_(1, 1200)_ = 5.49; *p* < 0.01]. The pairwise comparisons showed differences between retained and non-retained students (M_retained − Non_retained = 1.71, *p* < 0.01), with the latter sensing more support from their parents.

The second index (PS2) collects the perception of “respect and trust of both fathers and mothers,” showing a significative differences on the factor of being a retained student or not [*F*_(1, 1200)_ = 34.33; *p* < 0.001], but the interaction between factors is not significant. In the pairwise comparisons there are significant differences between native (M_retained − Non_retained = 1.82; *p* < 0.001) and immigrant students (M_retained − Non_retained = −1.21; *p* < 0.01), with non-retained students obtaining the highest score.

In the third factor, focused on the perception of “maternal support and encouragement when dealing with both school work and in problematic situations” (PS3), significant results were found [*F*_(1, 1200)_ = 10.55; *p* < 0.01] the factor relating to being a retained student or not, where only the native non-retained students [*F*_(1, 1200)_ = 10.55; *p* < 0.01] perceived more support and encouragement.

By analyzing their perception that “their father and mother clearly communicate their expectations” (PS4), no significant differences were observed in either the main effects or interaction. There is a greater presence of this index recorded in native non-retained students only within the pairwise comparison (M_retained − Non_retained = −0.57, *p* < 0.01).

Finally, the perception that their parents “care about their academic success” (PS5), is significant in the factor referred to being a retained student or not [*F*_(1, 1200)_ = 21.92, *p* < 0.001]. No significant differences were obtained in the interaction, although in the pairwise comparison, differences [*F*_(1, 1200)_ = 21.92; *p* < 0.001] regarding greater presence of this concern are noted, from the maternal and paternal side, in native non-retained students.

### Analysis of differences with respect to the assessment of the school environment

In this case significant differences were also found in four of the indices that make up this dimension and are summarized in Table 6.

**Table 6:** **Results of the analysis of variance (ANOVA) for the variables related to the assessment of school environment (NS-Native students; IS-Immigrant students)**.

**Factors**	**Ethno-cultural origin**		**Being retained**		**Interaction**		**Pairwise comparison**
	**Mean**	***F***	**Mean**	***F***	**Mean**	***F***	**Difference of means**
V1	NS (9.72)	*F*_(1, 1338)_ = 4.53^**^	No (9.22)	*F*_(1, 1338)_ = 40.91^***^	NS-No (9.21)	*F*_(1, 1338)_ = 5.25^**^	NS No-Yes = −2.05^**^
	IS (6.68)	η^2^ = 0.003, 1−β = 0.57	Yes (10.94)	η^2^ = 0.030, 1−β = 1.00	NS-Yes (11.26)	η^2^ = 0.004, 1−β = 0.63	IS No-Yes = −0.97^**^
					IS-No (9.25)		
					IS-Yes (10.22)		
V2	NS (6.35)	*F*_(1, 1338)_ = 8.80^**^	No (6.09)	*F*_(1, 1338)_ = 9.23^**^	NS-No (6.12)	*F*_(1, 1338)_ = 3.66^**^	NS No-Yes = −0.92^**^
	IS (6.02)	η^2^ = 0.007, 1−β = 0.84	Yes (6.76)	η^2^ = 0.007, 1−β = 0.86	NS-Yes (7.04)	η^2^ = 0.003, 1−β = 0.49	
					IS-No (5.93)		
					IS-Yes (6.13)		
V3	NS (7.30)	*F*_(1, 1338)_ = 1.65 ny	No (7.22)	*F*_(1, 1338)_ = 4.27 ny	NS-No (7.22)	*F*_(1, 1338)_ = 1.01 ny	
	IS (7.15)		Yes (7.38)		NS-Yes (7.51)		
					IS-No (7.19)		
					IS-Yes (7.02)		
V4	NS (5.39)	*F*_(1, 1338)_ = 7.91^**^	No (5.20)	*F*_(1, 1338)_ = 8.96^**^	NS-No (5.26)	*F*_(1, 1338)_ = 0.26 ny	NS No-Yes = −0.54^**^
	IS (5.07)	η^2^ = 0.006, 1−β = 0.80	Yes (5.64)	η^2^ = 0.007, 1−β = 0.86	NS-Yes (5.80)		
					IS-No (4.91)		
					IS-Yes (5.28)		
V5	NS (3.12)	*F*_(1, 1338)_ = 0.37 ny	No (3.02)	*F*_(1, 1338)_ = 15.74^***^	NS-No (2.99)	*F*_(1, 1338)_ = 1.03 ny	NS No-Yes = −0.51^**^
	IS (3.30)		Yes (3.50)	η^2^ = 0.012, 1−β = 0.98	NS-Yes (3.51)		
					IS-No (3.17)		
					IS-Yes (3.47)		

^*^p < 0.05,

** *p < 0.01*,

*** *p < 0.001*.

The first of these (V1), called “satisfaction with external feedback" refers to the reinforcements received from influential adults, when focused on their academic performance. Both factors and interaction were significant [*F*_(1, 1338)_ = 5.25, *p* < 0.01]. In the pairwise comparisons, significant differences were found in both native (M_retained − Non_retained = −2.05, *p* < 0.01) and immigrant students (M_retained − Non_retained = −0.97, *p* < 0.01), and the retained students from both groups are those who are said to receive greater external reinforcement, given the obtained academic results.

The same applies to the second index (V2), “satisfaction with school rules,” in which significant differences were observed in the factors [*F*_(1, 1338)_ = 8.80, *p* < 0.01 regarding the ethno-cultural origin and *F*_(1, 1338)_ = 9.23, *p* < 0.01 being a retained student or not], and the interaction between them [*F*_(1, 1338)_ = 3.66, *p* < 0.01]. As it can be noted in the pairwise comparison, the native retained students (M_retained − Non_retained = −0.92, *p* < 0.01) are those who show more satisfaction with their school's rules.

Table 6 shows that index V3, referring to “satisfaction and positive evaluation of the teaching styles,” does not show significant differences due to factors, or to the interaction between them. In the index comprising “satisfaction with the work performed in the classroom” (V4), significant differences were detected in both factors [*F*_(1, 1338)_ = 7.91, *p* < 0.01 for ethnic-cultural origin and *F*_(1, 1338)_ = 8.96, *p* < 0.01 for being a retained student or not], but not in the interaction between them. The native retained students (M_retained − Non_retained = 0.54, *p* < 0.01) are again those who, in the pairwise comparison, show greater satisfaction with the work performed in the classroom.

In the last factor (V5), whose content focuses on the “satisfaction with their relationships with their classmates and, in general, with the school,” the only factor showing significant differences [*F*_(1, 1338)_ = 15.74, *p* < 0.001] is being a retained student or not, although in the pairwise comparison, it was noted that the native retained students (M_retained − Non_retained = −0.51, *p* < 0.01) are the most satisfied with the treatment between peers and with the school in general.

### Regression analysis

Two stepwise logistic regressions were carried out to understand which of the analyzed variables can predict whether a student will be retained (1) or not (0). The obtained results are presented separately for native and immigrant students.

#### Predictors for immigrant students

First, the collinearity statistics were analyzed and tolerance values (T) ranging between 0.35 and 0.94 were observed; the variance inflation factor (VIF) obtained values between 1.06 and 2.85, indicating the lack of collinearity. On the other hand, using the Durbin–Watson test, a value of 1.93 was obtained, indicating independence of the residuals. Second, the Hosmer–Lemeshow statistic (χ^2^ = 10.81; *n* = 274; *df* = 8; *p* = 0.213) reflects a good fit of the model with an effect size that, according to Cohen (1988), is acceptable (Cox-Snell *R*^2^ = 0.44; Nagelkerke *R*^2^ = 0.59). Table 7 shows the results of the model including the regression coefficients, the Wald statistic and odds ratio Exp (B).

**Table 7:** **Results of logistic regression analysis for immigrant students**.

**Predictors**	***B***	**SE**	**Wald**	***p***	**Exp(B)**
Control 4	−0.049	0.026	3.60	0.050	0.95
Support 4	0.146	0.074	3.86	0.049	1.15
Rating 1	0.094	0.039	5.91	0.015	1.09
Spanish Language and Literature	−1.010	0.216	21.91	0.001	0.36
Age	1.950	0.305	40.85	0.001	7.03
Grade	−2.410	0.448	28.93	0.001	0.09
Siblings	0.301	0.141	4.55	0.033	1.35
**CLASSIFICATION TABLE**					
**Observed**	**Predicted**		**Correct %**		
	**Non-retained**	**Retained**			
Non-retained	128	24	84.2		
Retained	25	97	79.5		
Overall %			82.1		

The results show that a higher perceived paternal and maternal control over the hours devoted to daily study (Control 4) is associated with better academic performance (*B* = −0.049). In contrast, a greater concern of parents for academic success (Support 4) (*B* = 146) and satisfaction with the reinforcements they receive from influential adults (Rating 1) (*B* = 0. 094) are associated with being a retained student or not, which is why it is believed that both their family and teachers focus more on strengthening this group of retained students. The poor results obtained in Spanish Language and Literature (*B* = −1.01) are associated with retained students, who are generally older students (*B* = 1.95), with those in the lower grades (5th and 6th grade of Primary Education) showing a higher performance (*B* = −2.41) in this subject. Lastly, the students who are retained more times (*B* = 0.301) are those with a higher number of siblings.

The classification table shows that overall 82.1% of the participants are correctly classified and out of these the highest ranked are those who are not retained (84.2%) compared with retained students (79.5%).

#### Predictors for native students

The collinearity statistics were analyzed and tolerance values (T) ranging between 0.21 and 0.97 were observed; the variance inflation factor (VIF) obtained values between 1.02 and 4.76, indicating the lack of collinearity. On the other hand, using the Durbin–Watson test, a value of 1.86 was obtained, indicating independence of the residuals. Second, the Hosmer–Lemeshow statistic (χ^2^ = 9.29; *n* = 1080; *df* = 8; *p* = 0.318) indicates a good fit of the model with a good size effect (Cox-Snell *R*^2^ = 0.54; Nagelkerke *R*^2^ = 0.81). Table 8 shows the results of the model including the regression coefficients, the Wald statistic and odds ratio Exp (B).

**Table 8:** **Results of logistic regression analysis for native students**.

**Predictors**	***B***	**SE**	**Wald**	***p***	**Exp(B)**
Control 4	−0.121	0.039	9.85	0.002	0.88
Support 1	−0.048	0.024	4.05	0.044	0.95
Support 2	−0.066	0.023	8.3	0.004	0.94
Support 5	−0.096	0.045	4.37	0.034	0.91
Rating 1	0.175	0.033	28.88	0.001	1.19
Rating 3	−0.103	0.037	7.76	0.005	0.90
Spanish Language and Literature	−0.793	0.113	49.09	0.001	0.45
Mathematics	−0.417	0.106	15.62	0.001	0.66
Age	4.84	0.382	160.85	0.001	126.10
Grade	−5.72	0.521	120.85	0.001	0.003
**CLASSIFICATION TABLE**					
**Observed**	**Predicted**		**Correct %**		
	**Non-retained**	**Retained**			
Non-retained	783	29	96.4		
Retained	30	238	88.8		
Overall %			94.5		

In this case, the poor academic performance (retained students) is associated with poor paternal and maternal control over the hours devoted to daily study (Control 4) (*B* = −1.21), with a lower level of encouragement and assistance with school work and in problematic situations (Support 1) (*B* = −0.048), a low perception of respect and trust of parents (Support 2) (*B* = −0.066), a low perception of paternal and maternal concern regarding academic success (Support 5) (*B* = −0.096), with low grades in Spanish Language and Literature (*B* = −0.793) and Mathematics (*B* = −0.417) and a negative evaluation of the teaching styles of teachers for these subjects (Rating 3) (*B* = −0.103). At the same time, this group of students is the one receiving more external reinforcement from family and teachers when they are assessed (Rating 1) (*B* = 0.175). Contrary to what happens with the immigrant students, in this case, better academic performance (not being retained), lies with older students (*B* = 4.84); however, when analyzing the academic year, the 5th and 6th-grade Primary Education students (*B* = −5.72) are again those who obtain better academic results.

The classification table shows that 94.5% of the participants are correctly classified, out of whom the non-retained students (96.4%) are the most numerous compared with retained students (88.8%).

## Discussion

The analyses carried out allowed us to identify the variables related to family support and control, school satisfaction, and evaluation of the learning environment, which distinguish between the retained and non-retained native and immigrant students; and which of the selected variables clearly differentiate the two groups. The results are consistent with the scientific literature on the subject.

The data related to family control allow us to state that students with better performance (non-retained), both native and immigrant, perceived greater control from their parents, of their behavior outside the home. The native students with good performance are those who perceive, from both parents, greater control over the money spent, their school attendance, and the hours devoted to daily study, with this aspect being essential for their study habits. The results indicate the relationship between both parents' control and overall performance, especially in the case of Spanish children. It is well-known that the absence of psychological control, behavioral control, and autonomy support has positive effects on the academic achievement of adolescents and their behavioral adjustment (Barber et al., 2006; Wang et al., 2007). Santos Rego et al. (2016) consider that parental involvement (control and supervision) will be beneficial for the child when it supports the development of autonomy, when it focuses on the process and entails affection and positive beliefs; and, to the contrary, it may be detrimental if it is controlling, person-centered (emphasizing stable attributes) and characterized by negative affection and beliefs. Suárez et al. (2011) claimed that parental involvement in children's education had positive effects on their academic performance, as long as it was adequate, and involved support for the students.

Following the same line of research, Manrique et al. (2014) analyzed the association between negative parental control and positive parenting with achievement in spelling, arithmetic and reading in sixth-grade Primary Education students from Peru, concluding that the support and appropriate interactions can contribute to cognitive and psychological development during childhood. These authors also associated the high socioeconomic status with a lower negative parental control. Su et al. (2015) found in a sample of 310 Primary Education children from Germany that intrusive parental control was adversely associated with school performance.

Regarding study habits, recent studies have attempted to delve into the reasons why some students with good intellectual abilities perform worse on tests than others with lower abilities; these studies have confirmed the relationship between study habits and students' results in evaluation tests (Razia, 2015). More precisely, one of the variables that best predicts retention, both for native and immigrant students, is poor parental control over the hours devoted to daily study.

With respect to the support, the two groups of students with the best performance are again those who perceive greater respect and trust from their parents. The native students who were never retained feel supported and encouraged by both parents to carry out their school work and to address problematic situations, in addition to perceiving to a greater extent that their parents clearly communicate their expectations for them, and show a greater concern for their academic success.

Studies in other contexts and populations have confirmed similar results, showing the association between performance and family support. Bazán and Castellanos (2015) used a sample of Elementary Education 5th grade students from Mexico to conclude that perceived family support, especially from mothers, influences students' performance. In a sample of Primary Education students from Pakistan, Iqbal and Masrur (2010) examined the relationship between academic success and the educational support that children receive at home, and the effects of this support in their self-concept. The results showed that the parental support had a consistent and positive effect on academic success and self-concept (see Bean et al., 2003, 2006; Santana and Feliciano, 2011; Álvarez et al., 2015). According to Carbonero et al. (2015), students with a positive self-concept are effectively oriented toward learning (Cabanach et al., 2014).

The support variables that best predict retention in the case of native students are greater support and encouragement in school work and problematic situations, low perception of respect, and confidence from their parents, and the lack of parental concern for their academic successes. However, they receive greater external reinforcement (from family and teachers) when evaluated.

In the case of immigrant students, parents' biggest concern for academic success and the greatest satisfaction with the external reinforcements from influential adults (family and teachers) are also associated with retention.

Our results agree with those from other studies where it was proven that students with poorer performance received more family support (Chen, 2008; Bazán and Castellanos, 2015). This is explained by the fact that families and teachers are more focused on students who fail, in order to reinforce their knowledge. That is, families offer support when they realize that their children have learning difficulties. In any case, Alonso-Tapia and Simón (2012) suggest that immigrant students have a motivational profile associated with low self-esteem, which leads them to require a greater degree of external support than native students, from both teachers and their families, in order to help them overcome the lack of confidence.

On the other hand, the results show that the students most satisfied with school and the learning environment are precisely those who have poorer academic performance, especially among the native students. This suggests that these variables have no role in their poor performance.

When referring to immigrant students, it should be taken into account that our study involved a sample from Latin America. According to the results obtained in the study conducted by Santana et al. (2016), with Compulsory Secondary Education students of different origins, the level of support received by those from Latin America is higher than the one perceived by the other study groups. This may be associated with a higher educational level of the parents of this origin (Bazán et al., 2007; Lorenzo et al., 2012).

However, Schnell and Azzolini (2015) argue, contrary to most authors, on differences in achievement between immigrant and native students, that the parents' educational level plays a secondary role in explaining the performance differences, as adult immigrants in southern Europe generally have education levels similar to adult natives of those countries; thus, attention should be paid to the economic and material resources of these families. The same results were found in studies conducted in other countries (new immigration destinations) such as Finland (Harinen and Sabour, 2014) or Ireland (Fanning et al., 2011).

In short, it seems that the main differences focus on the individual dimensions (perception of family support and control) and, to a lesser extent, on the contextual dimensions (assessment of the school and its learning environments), which agrees with the results of other studies (Núñez et al., 2014). In any case, as argued by Santana et al. (2016), there have been few studies on the academic expectations, perceived family support, the decision-making process, and the life plan of immigrant students.

## Educational implications and study limitations

After performing this work, the need for studies that expand the sample size of participants should be considered, taking into account students of other ethno-cultural origins, with native languages other than Spanish. It would also be important to work with new variables, such as the time that these students have been living in Spain or levels of family involvement.

However, the results provide solid evidence aimed at designing programs for family involvement to help improve students' educational performance and contribute to the solution of school failure and dropouts, which is one of the main problems that education systems have to face today and, therefore, prevent the social exclusion of many young people in the future.

## Author contributions

AG and ML collected data, analyzed data, and wrote the paper. MF and MS analyzed data and wrote the paper.

### Conflict of interest statement

The authors declare that the research was conducted in the absence of any commercial or financial relationships that could be construed as a potential conflict of interest. The reviewer MI and the handling Editor declared their shared affiliation, and the handling Editor states that the process nevertheless met the standards of a fair and objective review.
